# Evaluation of the Repeatability and the Reproducibility of AL-Scan Measurements Obtained by Residents

**DOI:** 10.1155/2014/739652

**Published:** 2014-07-22

**Authors:** Mehmet Kola, Hikmet Duran, Adem Turk, Suleyman Mollamehmetoglu, Ahmet Kalkisim, Hidayet Erdol

**Affiliations:** Department of Ophthalmology, Faculty of Medicine, Karadeniz Technical University, 61080 Trabzon, Turkey

## Abstract

*Purpose*. To assess the repeatability and reproducibility of ocular biometry and intraocular lens (IOL) power measurements obtained by ophthalmology residents using an AL-Scan device, a novel optical biometer. *Methods*. Two ophthalmology residents were instructed regarding the AL-Scan device. Both performed ocular biometry and IOL power measurements using AL-Scan, three times on each of 128 eyes, independently of one another. Corneal keratometry readings, horizontal iris width, central corneal thickness, anterior chamber depth, pupil size, and axial length values measured by both residents were recorded together with IOL power values calculated on the basis of four different IOL calculation formulas (SRK/T, Holladay, and HofferQ). Repeatability and reproducibility of the measurements obtained were analyzed using the intraclass correlation coefficient (ICC). *Results*. Repeatability (ICC, 0.872*-*0.999 for resident 1 versus 0.905*-*0.999 for resident 2) and reproducibility (ICC, 0.916*-*0.999) were high for all biometric measurements. Repeatability (ICC, 0.981*-*0.983 for resident 1 versus 0.995*-*0.996 for resident 2) and reproducibility were also high for all IOL power measurements (ICC, 0.996 for all). *Conclusions*. The AL-Scan device exhibits good repeatability and reproducibility in all biometric measurements and IOL power calculations, independent of the operator concerned.

## 1. Introduction

Accurate calculation of biometric measurements and the power of the intraocular lens (IOL) to be implanted is an important issue in preventing residual errors that may remain after cataract surgery which is also described as a kind of refractive surgery [1, 2]. The importance of biometric measurements is even greater in patients scheduled to receive multifocal IOL or accommodating IOL implantation or refractive lens exchange [3]. Accurate IOL power calculation particularly depends on accurate measurement of corneal keratometry readings (*K*1, *K*2) and anterior chamber depth (ACD) and axial length (AL) values [4, 5]. Accurate measurement of pupil size (PS) and horizontal iris width (white-to-white [WTW]) values is another important factor that increases the success of cataract and refractive surgery [6–9].

The classic A-mode ultrasonic technique, which measures while in contact with the eye, and optical biometer devices that measure without touching the eye are currently used in AL measurement [5]. The ultrasonic technique has a number of disadvantages due to this ocular contact, such as corneal trauma and globe compression. Measurements also need to be coaxial with the ocular axis [4, 5, 10]. The entry into use of IOL Master (Carl Zeiss Meditec AG, Jena, Germany), which uses partial coherence interferometry (PCI), bestowed several advantages on AL measurement. This device was followed by the Lenstar LS 900 (Haag Streit AG, Bern, Switzerland) device that uses low-coherence optical reflectometry [1, 11]. These optical biometers preclude problems that may arise in association with ocular contact. Measurements are comparatively more repeatable and faster than those obtained using the ultrasonic technique [1, 4, 5, 10, 12, 13]. Due to the additional equipment and software in the devices, other parameters needed for IOL power calculation can be measured during the same session, and the power of the IOL needing to be installed during surgery can be calculated [1, 14].

Another optical biometer that has entered into use in recent years is the AL-Scan optical biometer (Nidek Co. Ltd., Japan). This uses an 830 nm super luminescent diode for AL measurement with PCI. It uses a light-emitting diode (LED) for corneal keratometry readings and WTW and PD assessment. The device uses the Scheimpflug principle to measure CCT and ACD values. The device is capable of performing IOL power calculation using various preprogrammed formulae [2, 14, 15]. The manufacturers state that no significant training is needed to use the AL-Scan, since the device's 3D autotracking and autoshot features perform biometric measurements as independently as possible of operator factors. The purpose of this study was to assess the reliability of measurements taken using AL-Scan by operators with no experience of optical biometry and thus to examine whether or not the device performs measurements independently of user factors, as the manufacturers claim.

## 2. Materials and Methods 

This prospective study, consisting of a case series, was performed at the Karadeniz Technical University, Faculty of Medicine, Department of Ophthalmology, Trabzon, Turkey. Local ethical committee approval was granted for the study protocol. Informed consent was obtained from all participants.

Patients presenting to the ophthalmology clinic with reduced vision were included in the study. Once detailed demographic data had been obtained, each participant was given a detailed ophthalmological examination. The best corrected visual acuity of each subject was recorded using the Snellen chart. Slit-lamp biomicroscopy, indirect fundus examination, and intraocular pressure measurements (using a noncontact tonometer) were subsequently performed.

The inclusion criteria were no additional ocular pathology other than refractive error and cataract and best-corrected visual acuity over 4/10 (on the Snellen scale) for both eyes. The exclusion criteria were previous ophthalmic surgery, an active eye pathology such as corneal diseases, pterygium, or dry eye that might affect ocular measurements, a history of contact lens use, vitreous opacity, optic disc anomaly, retinal diseases, inability to open the eyelid wide, poor ocular fixation, or a history of using topical/systemic medications for systemic diseases, which might interfere with the structure of the eye.

Two ophthalmology residents receiving specialist training at our university and with no previous experience of optical biometry were instructed regarding using the AL-Scan device. Each resident performed ocular biometry and IOL power measurements using the AL-Scan device, three times consecutively on both eyes. Measurements were performed independently, without assistance from any other individual, with the residents blind to one another. Between measurements, patients were asked to remove their heads from the device and to replace them for each new measurement. IOL power values obtained by each resident using corneal keratometry readings measured at a 2.4 mm diameter (*K*1, *K*2), horizontal iris width (white-to-white [WTW]), central corneal thickness (CCT), anterior chamber depth (ACD), pupil size (PS), and axial length (AL) values with four different IOL calculation formulas (SRK/T, Holladay, and HofferQ) were recorded.

### 2.1. Statistical Analysis

Measurement results were expressed as mean ± standard deviation. Statistical analyses were performed using SPSS 13.0.1 (SPSS, Chicago, IL, USA; license number 9069728, KTU, Trabzon, Turkey). Data normality was assessed using the Kolmogorov-Smirnov test. Repeatability of measurements obtained was calculated separately for each resident. Three consecutive measurement results obtained by each resident were employed for that purpose. Reproducibility was analyzed using the means of the three consecutive measurements taken by each resident. Intraclass correlation coefficient (ICC) values were calculated for repeatability and reproducibility analyses. In addition, variations in measurements taken by both residents in three separate sessions for each participant were examined using the Friedman test. Means of the three measurements performed by both residents for each participant were compared using the paired samples *t*-test and Pearson correlation analysis. *P* < 0.05 was considered significant.

## 3. Results 

One hundred twenty-eight eyes of 64 patients, 28 women and 36 men, were included in the study. Mean age of participants was 33.73 ± 11.87 years (22–64). Mean ocular biometry measurements obtained by both residents are shown in Table 1. Repeatability values for ocular biometry measurements were high for both residents (ICC, 0.872–0.999 for resident 1 versus 0.905–0.999 for resident 2).

Mean IOL power measurements performed by both residents using four different IOL calculation formulas are shown in Table 2. Repeatability values for IOL power measurements for both residents were again high (ICC, 0.981–0.983 for resident 1 versus 0.995–0.996 for resident 2). Variations in IOL power measurements performed by both residents were similar (*P* > 0.05 for all).

The means of the three consecutive ocular biometry measurements performed by both residents are shown in Table 3. Analysis of these values again revealed quite high reproducibility (ICC, 0.916–0.999).

**Figure 1:**
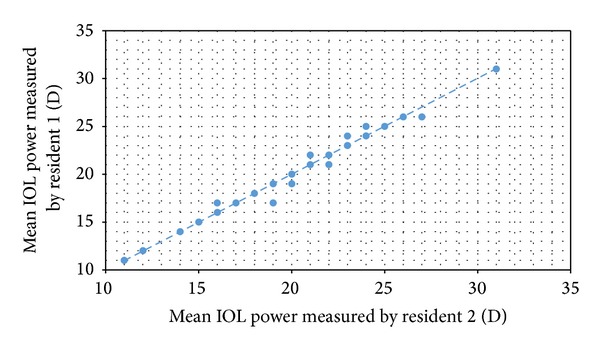
Correlation of mean IOL power measurements performed by both residents using the SRK/T formula.

Means of the three consecutive power IOL measurements performed by both residents are given in Table 4. Analysis of these values again revealed high reproducibility (ICC, 0.996 for all). Mean IOL power measurements performed by both residents exhibited a close correlation (*r* = 0.996, *P* < 0.0001 for the SRK/T formula (Figure 1); *r* = 0.996, *P* < 0.0001 for the Holladay formula; and *r* = 0.996, *P* < 0.0001 for the HofferQ formula), and variations in these measurements were again similar (*P* > 0.05 for all).

The short-term training effect on measurements obtained by the residents was also examined using IOL power measurements taken in the first 10 and last 10 patients. IOL power measurements performed according to the SRK/T formula were selected for this purpose. ICC values for IOL power repeatability in the first 10 patients were 0.994 (0.988–0.998) for the first resident and 0.995 (0.99–0.998) for the second resident. The values in IOL power measurements obtained in the last 10 patients were 0.998 (0.996–0.999) for the first resident and 0.997 (0.994–0.999) for the second resident. ICC values reflecting IOL power measurement reproducibility were 0.997 (0.992–0.999) in the first 10 patients and 0.999 (0.997–1.000) in the last 10 patients. These results show that the effect of short-term training effect on resident measurements was clinically insignificant.

## 4. Discussion

Generally, the most crucial steps in IOL power calculation are corneal radius and AL measurements. A 1 mm measurement error in corneal radius results in a refractive error of approximately 5.7 D, while a 1 mm error in AL measurement results in an approximately 2.7 D refractive error [5]. It is therefore very important for biometric measurements to be performed accurately. With the entry into use of optical biometers, the reliability of these measurements has increased in comparison with older, classic techniques. Inability to measure the postoperative effective lens position is the most important source of error in IOL power measurement using the modern optical biometric technique [5].

Various studies in the literature have compared optical biometers [4, 10, 16–18]. However, the number of studies concerning AL-Scan, a novel biometer, is still inadequate. Huang et al. [15] studied 68 eyes and reported that the AL-Scan device produced highly repeatable and reproducible measurements, with the exception of WTW and PD measurements. Huang et al. [15] also reported that AL-Scan exhibited good agreement with IOL Master, again with the exception of WTW measurements [15]. In contrast to that study, like the other measurements, the WTW and PD measurements obtained with AL-Scan in our study were highly repeatable and reproducible. These differences between the two studies in terms of WTW and PD measurements may derive from individual factors in the participants in both, such as iris color and cataract density, or differences such as intensity of illumination in the environment where measurements took place. Another possible reason may be that the time intervals between consecutive measurements were not the same. In a study of 50 eyes, Kaswin et al. [2] determined that the AL-Scan device produced results compatible with IOL Master in terms of both biometric measurements (AL, K, and ACD measurements) and IOL power calculation. That study determined insignificant differences between the two devices of 0.01 ± 0.004 mm in AL measurements, 0.17 ± 0.03 D in keratometry measurements, and 0.13 ± 0.04 mm in ACD measurements. The study also reported insignificant differences between the two devices of 0.021 ±  0.048 D in IOL power values calculated according to the Haigis formula and of 0.029 ± 0.037 D in IOL power values calculated according to the SRK/T formula [2].

In another study of 137 eyes conducted by Srivannaboon et al. [14], agreement between biometric measurements obtained with AL-Scan and IOL Master was quite high, apart from WTW values. In that same study, ICC values reflecting repeatability in biometric measurements obtained with AL-Scan were reported as *K* at 2.4 mm ICC = 0.999, AL ICC = 1.000, ACD ICC = 0.999, WTW ICC = 0.945, and IOL power with the Holladay 1 formula ICC = 0.999. ICC values reflecting reproducibility in biometric measurements obtained with AL-Scan were reported as *K* at 2.4 mm ICC = 0.998, AL ICC = 0.999, ACD ICC = 0.999, WTW ICC = 0.873, and IOL power with the Holladay 1 formula ICC = 0.998 [14]. In our study, ICC values reflecting repeatability and reproducibility in AL-Scan measurements obtained by two residents with no previous experience were close to the values reported by Srivannaboon et al. [14], thus confirming the excellent repeatability and reproducibility characteristics of the device.

The inclusion of both eyes due to the low number of subjects in this study may be regarded as a limitation. However, since the aim of the study was not data collection, but comparison of consecutive measurements, this can be overlooked. Another limitation is that the AL-Scan measurements taken by two inexperienced residents were not compared with those performed by an experienced operator.

In conclusion, this is the first study to test operator experience in calculating biometric measurements obtained with an AL-Scan device and IOL power. All measurements performed by two inexperienced operators using an AL-Scan device exhibited quite high repeatability and reproducibility. The AL-Scan device is easy and comfortable to use and performs rapid and reproducible measurements.

## Figures and Tables

**Table 1:** Comparison of three consecutive biometric measurements taken by both ophthalmology residents (Repeatability).

Parameter	Resident	1st measurement Mean ± SD (Min–Max)	2nd measurement Mean ± SD (Min–Max)	3rd measurement Mean ± SD (Min–Max)	ICC (Confidence interval)
*K*1 (D)	1	43.03 ± 1.29 (40.18–45.61)	43.02 ± 1.32 (40.08–46.30)	43.04 ± 1.29 (40.23–45.67)	0.995 (0.993–0.996)
	2	43.01 ± 1.32 (40.08–45.79)	43.02 ± 1.32 (40.13–45.73)	43.01 ± 1.32 (39.94–45.67)	0.993 (0.99–0.995)

*K*2 (D)	1	43.92 ± 1.40 (40.37–47.74)	43.93 ± 1.40 (40.42–47.20)	43.95 ± 1.39 (40.37–47.14)	0.993 (0.991–0.995)
	2	43.88 ± 1.41 (40.52–47.07)	43.92 ± 1.38 (40.37–47.20)	43.91 ± 1.38 (40.37–47.27)	0.988 (0.984–0.991)

WTW (mm)	1	12.13 ± 0.44 (10.7–13.2)	12.13 ± 0.44 (10.7–13.2)	12.11 ± 0.45 (10.8–13.2)	0.937 (0.917–0.954)
	2	12.12 ± 0.44 (10.8–13.2)	12.11 ± 0.44 (10.7–13.2)	12.12 ± 0.43 (10.7–13.2)	0.96 (0.947–0.97)

CCT (*μ*)	1	554.88 ± 37.84 (479–677)	555.37 ± 37.32 (482–676)	556.5 ± 37.03 (480–676)	0.97 (0.96–0.978)
	2	553.84 ± 35.84 (478–674)	554.91 ± 37.04 (482–673)	555.53 ± 37.12 (481–677)	0.96 (0.947–0.971)

ACD (mm)	1	3.65 ± 0.38 (2.8–4.67)	3.66 ± 0.38 (2.81–4.67)	3.66 ± 0.38 (2.8–4.68)	0.995 (0.993–0.996)
	2	3.65 ± 0.39 (2.79–4.67)	3.65 ± 0.38 (2.8–4.69)	3.65 ± 0.38 (2.8–4.69)	0.995 (0.993–0.996)

PS (mm)	1	5.91 ± 1.23 (3.4–8.3)	5.72 ± 1.26 (3.2–8.4)	5.55 ± 1.26 (3.1–8.3)	0.872 (0.832–0.905)
	2	5.91 ± 1.21 (3.3–8.3)	5.7 ± 1.23 (3.2–8.4)	5.63 ± 1.22 (3–8.3)	0.905 (0.875–0.929)

AL (mm)	1	23.66 ± 1.06 (20.89–27.23)	23.66 ± 1.05 (20.9–27.2)	23.66 ± 1.07 (20.9–27.23)	0.999 (0.998-0.999)
	2	23.66 ± 1.06 (20.89–27.23)	23.66 ± 1.06 (20.91–27.22)	23.66 ± 1.05 (20.91–27.23)	0.999 (0.998-0.999)

**Table 2:** Comparison of three consecutive IOL power measurements taken by both ophthalmology residents (Repeatability).

Parameter	Resident	1st measurement Mean ± SD (Min–Max)	2nd measurement Mean ± SD (Min–Max)	3rd measurement Mean ± SD (Min–Max)	ICC (Confidence interval)
SRK/T (D)	1	21.27 ± 2.96 (10.98–30.86)	21.27 ± 2.93 (11.1–30.9)	21.28 ± 2.95 (10.95–30.93)	0.983 (0.977–0.988)
	2	21.32 ± 2.91 (11.53–30.86)	21.28 ± 2.96 (11.22–30.72)	21.28 ± 2.94 (11.02–31)	0.996 (0.995–0.997)
	Difference	−0.045 ± 0.286 (−1.73–0.66)	−0.006 ± 0.189 (−0.66–0.6)	0.039 ± 0.636 (−2.85–5.78)	*P* = 0.379*

Holladay (D)	1	21.25 ± 3.05 (10.46–31.35)	21.25 ± 3.03 (10.59–31.41)	21.26 ± 3.05 (10.43–31.44)	0.983 (0.977–0.987)
	2	21.3 ± 3.01 (11.06–31.35)	21.25 ± 3.05 (10.72–31.21)	21.26 ± 3.04 (10.5–31.53)	0.996 (0.994–0.997)
	Difference	−0.052 ± 0.308 (−1.87–0.63)	−0.006 ± 0.21 (−0.71–0.64)	0.042 ± 0.662 (−2.7–6.11)	*P* = 0.296*

HofferQ (D)	1	21.20 ± 3.18 (10.58–31.84)	21.2 ± 3.16 (10.7–31.9)	21.22 ± 3.18 (10.53–31.94)	0.981 (0.974–0.986)
	2	21.26 ± 3.14 (11.23–31.84)	21.21 ± 3.18 (10.84–31.69)	21.22 ± 3.16 (10.61–32.03)	0.995 (0.994–0.996)
	Difference	−0.059 ± 0.338 (−2.14–0.67)	−0.006 ± 0.228 (−0.76–0.68)	0.044 ± 0.728 (−2.74–6.83)	*P* = 0.397*

*Friedman test.

**Table 3:** Comparison of three consecutive biometric measurement means obtained by both ophthalmology residents (Reproducibility).

Parameter	Resident	Mean ± SD (Min–Max)	ICC (Confidence interval)
*K*1 (D)	1	43.05 ± 1.3 (40.16–45.82)	0.997 (0.995–0.998)
	2	43.01 ± 1.32 (40.05–45.69)	

*K*2 (D)	1	43.95 ± 1.39 (40.39–47.36)	0.996 (0.994–0.997)
	2	43.91 ± 1.38 (40.42–47.16)	

WTW (mm)	1	12.12 ± 0.44 (10.73–13.2)	0.979 (0.97–0.985)
	2	12.12 ± 0.43 (10.73–13.2)	

CCT (*μ*)	1	556.12 ± 37.19 (480.33–675)	0.99 (0.986–0.993)
	2	554.76 ± 36.18 (480.33–673.33)	

ACD (mm)	1	3.66 ± 0.38 (2.8–4.67)	0.997 (0.996–0.998)
	2	3.65 ± 0.38 (2.8–4.68)	

PS (mm)	1	5.72 ± 1.21 (3.23–8.3)	0.916 (0.882–0.941)
	2	5.76 ± 1.18 (3.27–8.33)	

AL (mm)	1	23.66 ± 1.06 (20.9–27.22)	0.999 (0.999-1)
	2	23.66 ± 1.06 (20.9–27.23)	

**Table 4:** Comparison of IOL power measurement means obtained by both ophthalmology residents (Reproducibility).

Parameter	Resident	Mean ± SD (Min–Max)	ICC (Confidence interval)
SRK/T (D)	1	21.25 ± 2.93 (11.01–30.9)	0.996 (0.995–0.998)
	2	21.29 ± 2.93 (11.26–30.86)	
	Difference	−0.0039 ± 0.247 (−0.91–2.01)	*P* = 0.861*

Holladay (D)	1	21.22 ± 3.03 (10.49–31.4)	0.996 (0.995–0.997)
	2	21.27 ± 3.03 (10.76–31.36)	
	Difference	−0.0044 ± 0.26 (−0.85–2.13)	*P* = 0.849*

HofferQ (D)	1	21.17 ± 3.16 (10.6–31.89)	0.996 (0.994–0.997)
	2	21.23 ± 3.16 (10.89–31.85)	
	Difference	−0.0059 ± 0.286 (−0.85–2.37)	*P* = 0.816*

*Paired samples *t* test.
